# Membrane Contact Sites and Organelles Interaction in Plant Autophagy

**DOI:** 10.3389/fpls.2020.00477

**Published:** 2020-04-24

**Authors:** Hao Ye, Changyang Ji, Rongfang Guo, Liwen Jiang

**Affiliations:** ^1^School of Life Sciences, Centre for Cell and Developmental Biology and State Key Laboratory of Agrobiotechnology, The Chinese University of Hong Kong, Hong Kong, China; ^2^College of Horticulture, Fujian Agriculture and Forestry University, Fuzhou, China; ^3^CUHK Shenzhen Research Institute, Shenzhen, China

**Keywords:** autophagy, autophagosome, MCS, ORP, NBR1

## Abstract

Autophagy is an intracellular trafficking and degradation system for recycling of damaged organelles, mis-folded proteins and cytoplasmic constituents. Autophagy can be divided into non-selective autophagy and selective autophagy according to the cargo specification. Key to the process is the timely formation of the autophagosome, a double-membrane structure which is responsible for the delivery of damaged organelles and proteins to lysosomes or vacuoles for their turnover. Autophagosomes are formed by the closure of cup-shaped phagophore which depends on the proper communication with membrane contributors. The endoplasmic reticulum (ER) is a major membrane source for autophagosome biogenesis whereby the ER connects with phagophore through membrane contact sites (MCSs). MCSs are closely apposed domains between organelle membranes where lipids and signals are exchanged. Lipid transfer proteins (LTPs) are a large family of proteins including Oxysterol-binding protein related proteins (ORP) which can be found at MCSs and mediate lipid transfer in mammals and yeast. In addition, interaction between autophagosomes and other organelles can also be detected in selective autophagy for selection and degradation of various damaged organelles. Selective autophagy is mediated by the binding of a receptor or an adaptor between a cargo and an autophagosome. Here we summarize what we know about the MCS between autophagosomes and other organelles in eukaryotes. We then discuss progress in our understanding about ORPs at MCSs in plants and the underlying mechanisms of selective autophagy in plants with a focus on receptors/adaptors that are involved in the interaction of the autophagosome with other cytoplasmic constituents, including the Neighbor of BRCA1 gene 1 (NBR1), ATG8-interacting protein 1 (ATI1), Regulatory Particle Non-ATPase 10 (RPN10), and Dominant Suppressor of KAR2 (DSK2).

## Introduction

Macroautophagy (hereinafter autophagy) is an evolutionary conserved mechanism for the degradation of damaged or unwanted cytoplasmic materials in eukaryotes. During autophagy, a double membrane-bounded organelle, termed the autophagosome, is formed to engulf unwanted cytoplasmic materials and subsequently fuses with the lysosome/vacuole leading to the degradation of the cargo (Mizushima and Komatsu, 2011; Cui et al., 2020). Over the past decades, the mechanism of autophagy has been extensively studied in yeast and mammal for its important roles in not only subcellular degradation and quality control, but also in terms of stress response and human disease. For plants autophagy, as a major degradation process, is vital to plant development, immune response, starvation and stress response (Han et al., 2011; Cui et al., 2019).

During autophagy, the phagophore is formed at diverse membrane sites such as the endoplasmic reticulum (ER), ER-mitochondria contact sites, the ER-Golgi intermediate compartment, the plasma membrane (PM) and the Golgi apparatus (Axe et al., 2008; Hayashi-Nishino et al., 2009; Ravikumar et al., 2010; Ge et al., 2013; Hamasaki et al., 2013). Then the phagophore expands and enwraps targeted cytoplasmic materials to form a cup-shaped structure. The subsequent closure of this structure leads to the formation of a double-membrane-bound, sequestering autophagosome. Autophagy can be either selective or non-selective. Non-selective autophagy is a cellular response to nutrient deprivation and involves random engulfment of cytoplasm into autophagosomes for degradation and reuse, whereas selective autophagy removes unwanted or damaged subcellular compartments via specific interaction of receptors with their cargos and autophagosomal membrane (Gatica et al., 2018).

The ER membrane contact site (MCS) plays a central role in autophagosome formation because it mediates the transportation of essential lipids to autophagic membrane from the ER. In addition, autophagosomes also interact and communicate with other organelles in selective autophagy, but the molecular mechanisms underlying autophagosome-organelle communication remain largely elusive. In this review, we will discuss models of autophagosome-organelles interaction from the perspectives of MCS and selective autophagy receptors in plants.

## Membrane Contact Sites in Autophagosome Biogenesis

**FIGURE 1:**
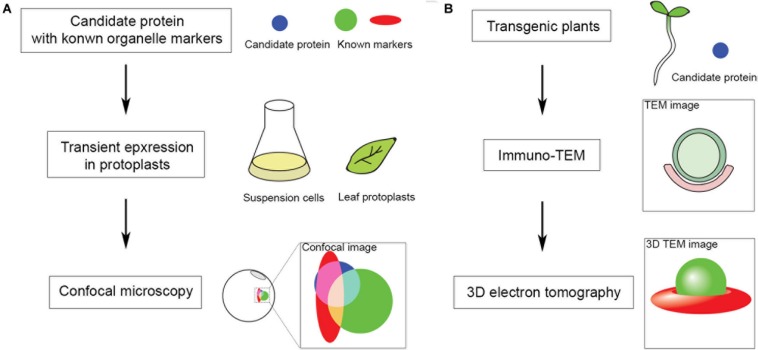
Fast screening approach to identify protein candidates localized at organelle MCS for subsequent ultrastructural study in Arabidopsis. MCS markers are mostly unknown in Arabidopsis, hence, to start with: **(A)** Fluorescent tagged candidates are co-expressed with known organelle markers in Arabidopsis protoplasts for subsequent confocal imaging analysis. **(B)** Positive candidates identified in panel **(A)** are further transformed and expressed in transgenic Arabidopsis seedlings for subsequent immune gold-TEM and 3D electron tomographic (ET) analysis.

**FIGURE 2:**
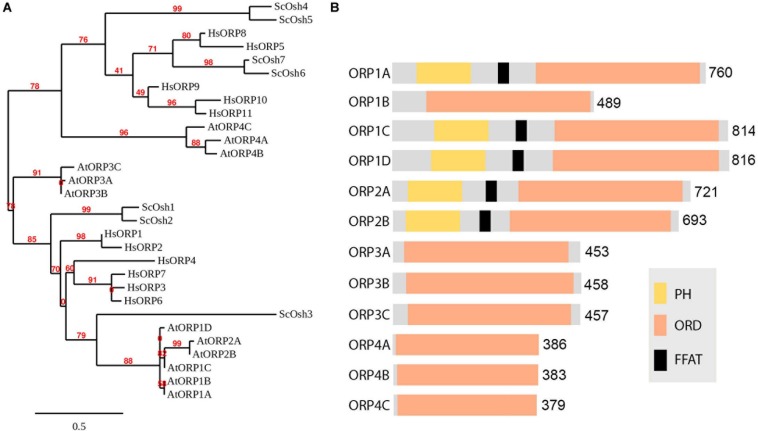
Phylogenetic analysis of the Arabidopsis ORP family. **(A)** Phylogenetic tree of Oxysterol-binding Protein Related Proteins (ORPs) from Homo sapiens (Hs), Saccharomyces cerevisiae (Sc) and Arabidopsis thaliana (At) and viewed with MEGA4 program. Bar = 0.5. **(B)** Typical ORP contains both oxysterol-binding related domain ORD and PH domains, and a FFAT motif in between. Some potential VAP relevant core FFAT elements are not shown in the diagram as it can be very short which is commonly found in the sequence.

The ER is one of the most important endomembranes working as a central hub in subcellular communication and material preparation that occupies a large amount of cytoplasmic volume and extends throughout the cell (Wu et al., 2018). Almost all organelles have been reported to be in contact with the ER for signal transmission and material exchange. The ER is also reported to be in contact with autophagic structures and is important for autophagosome formation (Zeng et al., 2019a). Before separating from the ER, the phagophore is kept in contact with the ER by the interaction of the integral ER protein VAMP-associated proteins (VAP) A and B (VAPs) and autophagosomal core machinery protein WD repeat domain phosphoinositide-interacting protein 2 (WIPI2/ATG18) (Zhao et al., 2018). Numerous studies have shown that VAPs mediate MCSs between ER and other organelles cooperating with proteins containing two phenylalanines (FF) in an acidic tract (FFAT) motif (Murphy and Levine, 2016). Most FFAT proteins interact with lipids for positioning onto non-ER membranes, responsible being lipid transfer proteins (LTPs). Oxysterol-binding protein (OSBP)-related proteins (ORPs) are well-studied LTPs in maintaining various MCSs and transferring lipids (Hammond and Pacheco, 2019). Although the FFAT motif is absent in some ORPs, most of them are located at multiple MCSs (Du et al., 2018). Research shows that ORP1L localizes and forms ER-autophagosome contact sites interacting with VAPA under low-cholesterol conditions and governs the autophagosome/lysosome fusion (Wijdeven et al., 2016). This is an important step to involve LTP in ER-autophagosome contact studies, as lipids are critical in forming the basis of membrane structure, signaling in membrane dynamics and recruitment of functional proteins (Enrique Gomez et al., 2018). As far as we know, the ER is the major organelle for lipid biogenesis as it can generate and recruit all the materials needed. It is conceivable that signaling/constituent lipids are synthesized at the ER membrane, and LTP may be the one that assists in lipid transfer at MCSs. In plants, homologs of Arabidopsis VAPs named VAP27s were identified through their conserved major sperm domain (MSD). VAP27 localizes to the ER as well as ER-PM contact site (EPCS), and VAP27-1 has been shown to function in cytoskeletal organization and endocytosis (Wang et al., 2014, 2016b; Siao et al., 2016; Stefano et al., 2018). It is also expected to be important in forming ER-autophagic structure contacts. It has long been believed that the ER is the platform and important membrane source for autophagosome biogenesis, however, in plants there has been little evidence for the existence of ER-autophagosome contact sites (Zhuang et al., 2018). Indeed, preliminary transmission electron microscope (TEM) studies on Arabidopsis root cells showed the occasional connection and contacts of ER and autophagic structures during autophagy, however, the tethering molecules of membrane contacts and functions remain elusive. A fast screening approach can be used to identify protein candidates localized at MCSs for subsequent TEM-based structural characterization of MCSs (Figure 1). Further studies will focus on the function and membrane tethering of these protein candidates at the MCS. It can be predicted that VAP27s may be involved in forming and maintaining the membrane tethering to work as membrane anchors in ER-autophagic structure contact sites. One of the FFAT proteins [including variants defined as FFAT-like (FFAT-L) motif containing proteins (Chew et al., 2013)] works as the counterpart of VAP in membrane tethering is the ORP family. 12 Arabidopsis ORPs have been identified according the conserved oxysterol-binding related domain (ORD) (Figure 2; Skirpan et al., 2006). A few studies have shown the importance of selected ORPs in sterol biogenesis and MCSs, however, their function in lipid regulation and membrane trafficking as well as in plant development is poorly understood (Saravanan et al., 2009; Umate, 2011; Barajas et al., 2014; Yao and Xue, 2018).

Most ER-membrane contacts have multiple tethering molecules. In yeast and mammalian cells, in addition to the possible VAP-FFAT protein complex, an Atg18-Atg2 complex was reported to be the tethering pair bridging the ER and the nascent autophagosome. In yeast cells, the Atg18-Atg2 complex localizes both to the edge of phagophore as well as the ER subdomain via Atg18-phosphatidylinositol 3-phosphate (PI3P) and Atg2-Atg9 binding (Obara et al., 2008; Suzuki et al., 2013; Gómez-Sánchez et al., 2018). Loss of proper localization of Atg18 by disrupting PI3P interaction reduces autophagic activity (Obara and Ohsumi, 2011; Kobayashi et al., 2012). Atg2, a conserved core autophagic protein of around 200 kDa, is found on the ER membrane but its function has been somewhat of a mystery. Recently, numerous functional and structural studies have shown that Atg2 mediates membrane tethering and phospholipid transport *in vitro*, suggesting that Atg2 functions as an LTP for phagophore positioning and expansion (Kotani et al., 2018; Osawa and Noda, 2019; Osawa et al., 2019; Zhang, 2020). Plants often have multiple homologs of proteins that mammal and yeast do not, which makes research more difficult to perform. For instance, there are 8 ATG18 homologs in Arabidopsis named ATG18a-h found with potential diverse functions in autophagosome formation. ATG2 and ATG18a function together as a complex in autophagosome expansion and maturation downstream of the initiation of autophagy by ATG11 and ATG9 (Kang et al., 2018). Although the precise functions of ATG2 and ATG18s remain unknown in plants, they may play other roles in addition to mediating the formation of autophagosomal MCS (Kang et al., 2018; Huang et al., 2019).

In mammalian cells, a multispanning ER-resident protein known as vacuole membrane protein 1 (VMP1) has been reported to localize in close proximity to various organelles such as mitochondria, lipid droplets and endosomes (Molejon et al., 2013). VMP1 plays an important role in autophagosome formation and progression. During autophagy, VMP1 is enriched at the ER subdomains to recruit and activate PI3P kinase (PI3K) complex to prepare the key lipids for autophagosome formation (Molejon et al., 2013; Tábara and Escalante, 2016). Depletion of VMP1 results in enhanced interaction between the autophagy core machinery proteins WIPI2 and Unc-51 like autophagy activating kinase (ULK1) complex, thus blocking the disassociation of phagophore with the ER (Zhao et al., 2018). Notably, VMP1 also controls the formation of MCSs via interacting with SERCA (sarco/endoplasmic reticulum calcium ATPase, the type P ATPase pump that transports calcium ions) and promoting its activity (Zhao et al., 2018).

Other ER MCSs also involved in the biogenesis of autophagosome in mammalian cells, such as the ER-mitochondria MCS (Hamasaki et al., 2013) and ER-PM contact site (Nascimbeni et al., 2017). During starvation-induced autophagy, ER residential protein syntaxin 17 (STX17) is redistributed at ER-mitochondria MCS and subsequentially recruits VPS34 PI3K complex to generate PI3P for autophagosome formation (Hamasaki et al., 2013). At ER-PM contact site, the tethering molecules extended synaptotagmins (E-syts) recruit VPS34 via interacting with VMP1 for autophagosome formation (Nascimbeni et al., 2017). In plants, several recent studies suggested the possible involvement of MCS proteins in autophagy-related stress response and autophagosome formation (Pérez-Sancho et al., 2015; Sancho et al., 2015; Wang et al., 2016a, 2019; Wang and Hussey, 2019), however, the molecular mechanisms underlying MCSs function in regulating autophagosome biogenesis in plants remain elusive.

## Selective Autophagy of Organelles and Receptors

Besides autophagosomal MCSs, autophagosomes can also interact with other organelles via receptors during organellophagy, the clearance of organelles via selective autophagy. Organellophagy is critical for maintaining cellular homeostasis by sequestering and eliminating organelles to adjust to environmental cues. Mitochondria, peroxisomes, lysosomes, nucleus, ER, and chloroplasts have been identified as cargos of organellophagy, the processes being called mitophagy (Ashrafi and Schwarz, 2013), pexophagy (Hutchins et al., 1999), lysophagy (Hung et al., 2013), nucleophagy (Roberts et al., 2003), ER-phagy (or reticulophagy) (Bernales et al., 2007; Zeng et al., 2019b), and chlorophagy (Izumi and Nakamura, 2017). In the stress response, cells control the integrity and numbers of these organelles and recycle the nutrients, remove damaged ones and control the quality. Organellophagy entails recognition and degradation of a specific cargo. This involves an autophagy receptor that bridges the autophagic membrane and cargo for sequestering and degradation. These selective autophagic pathways have been extensively studied in yeast and mammalian cells, however, research in plants is still in its infancy.

The chloroplast is a well-known plant organelle providing plants with energy. Multiple pathways of chlorophagy have been reported for the degradation of partial or whole chloroplasts during various conditions such as photodamage, starvation or senescence (Wada et al., 2009; Izumi and Nakamura, 2018). During chlorophagy, the entire photodamaged chloroplasts were transported by autophagosome to the vacuole for degradation (Izumi et al., 2017). Chloroplast/plastid contents can also form rubisco-containing bodies (Ishida et al., 2008), ATG8-INTERACTING PROTEIN 1-GFP labeled plastid-associated (ATI-PS) bodies (Michaeli et al., 2014), or small starch granule-like structures (SSGL) (Wang et al., 2013) and then wrapped by autophagosomes for subsequent degradation in vacuoles. Studies have shown that ATI is linked to ATG8 and plastid proteins to function as a receptor (Michaeli et al., 2014), but for the whole chloroplast degradation, the nature of the receptor is still not clear.

Other than being a platform for autophagosome biogenesis, ER fragments can act as a specific cargo for selective autophagy under conditions of ER stress. This is known as ER-phagy. A reasonable explanation as to why part of the ER is engulfed by autophagosomes is to eliminate ER luminal unfolded proteins or aggregates created during ER stress. A recent study in Arabidopsis suggests AtSec62a is the receptor mediating the ER-phagy. This is a translocon protein with three transmembrane domains (TMDs) and 2 ATG8-interacting motifs (AIMs) facing the cytosol. AtSec62 is engulfed by autophagosomes only when ER stress is induced, and this pathway depends on lipidated ATG8. Loss-of-function of AtSec62 fails to give rise to ER-phagy and plants exhibit ER stress hyper-sensitivity, indicating that AtSec62 may function as an ER-phagy receptor to mediate misfolded protein clearance as its homologues do in mammalian cells (Deshaies and Schekman, 1989; Fumagalli et al., 2016; Hu et al., 2019).

Selective autophagy receptors for other organelles such as mitochondria and peroxisome have been well-studied in mammals and yeast. For example, five receptors including NIX/BNIP3L (Zhang and Ney, 2008), p62/SQSTM1 (Geisler et al., 2010), FUNDC1 (Liu et al., 2012), BNIP3 (Zhang and Ney, 2009), and optineurin (Lazarou et al., 2015) were shown to involve in the degradation of mammalian mitochondria, whereas one receptor Atg32 (Kanki et al., 2009) was responsible for the turnover of mitochondria in yeast. In addition, the mammalian p62 (Kim et al., 2008) and NBR1 (Deosaran et al., 2013) as well as the yeast Atg30 (Farré et al., 2008) and Atg36 (Motley et al., 2012) were shown to function in pexophagy. However, relatively little is known about the specific receptors in plant selective autophagy albeit its extensive study in recent years. Taken together, selective autophagy is a sophisticated machinery with multiple receptors and their specific cargos (Table 1).

**TABLE 1:** Cargo and receptor of selective autophagy in yeast, animals and plant.

Cargo	Receptor in plant	Receptor in mammal	Receptor in yeast
Endoplasmic Reticulum	Sec62 (Hu et al., 2019)	FAM134B (Khaminets et al., 2015), Bnip3/Nix (Hanna et al., 2012)	Atg39 (Mochida et al., 2015), Atg40 (Mochida et al., 2015)
Mitochondria	N.C.	NIX/BNIP3L (Zhang and Ney, 2008), p62/SQSTM1 (Geisler et al., 2010), FUNDC1 (Liu et al., 2012), BNIP3 (Zhang and Ney, 2009), OPTN (Lazarou et al., 2015)	Atg32 (Kanki et al., 2009)
Peroxisome	N.C.	NBR1 (Deosaran et al., 2013), p62 (Kim et al., 2008)	Atg30 (Farré et al., 2008), Atg36 (Motley et al., 2012)
Aggregate	NBR1 (Jung et al., 2019)	ALFY (Simonsen et al., 2004), NBR1 (Kirkin et al., 2009), p62 (Pankiv et al., 2007)	N.C.
Nuclear	N.C.	N.C.	Atg39 (Mochida et al., 2015)
Ape1, Ams1	N.C.	N.C.	Atg19/Cvt19 (Scott et al., 2001), Atg34 (Suzuki et al., 2010)
Pathogen/Virus	NBR1 (Hafren et al., 2017), Joka2 (Dagdas et al., 2018)	OPTON (Wild et al., 2011), NDP52 (Thurston et al., 2009), p62 (Zheng et al., 2009)	N.C.
Plastid	ATI (Michaeli et al., 2014)	N.C.	N.C.
BES1	DSK2 (Nolan et al., 2017)	N.C.	N.C.
26S proteasome	RPN10 (Marshall et al., 2015)	N.C.	N.C.
FLS2	ORM (Yang et al., 2019)	N.C.	N.C.

*ATG, Autophagy-Related Gene; ATI, ATG8-Interacting Protein1; ALFY, Autophagy-Linked FYVE Protein; DSK2, Dominant Suppressor of KAR2; FLS2, Flagellin-sensing 2; NBR1, Neighbor of BRCA1 Gene 1; N.C., Not characterized; NDP52, Nuclear Dot Protein 52 kDa; NIX, NIP-like protein x; ORM, Orosomucoid; RPN10, Regulatory Particle Non-ATPase 10; OPTN, Optineurin.*

## The Plant Cargo Receptor NBR1

The ubiquitin-binding protein p62 was the first identified receptor in selective autophagy in mammalian cells (Danieli and Martens, 2018). Genetic studies have revealed that p62 gene mutation is related to several human diseases including amyotrophic lateral sclerosis (ALS) and frontotemporal dementia (FD) (Fecto et al., 2011; Goode et al., 2016). p62 contains several conserved domains and motifs mediating its function as an autophagic cargo receptor. The N-terminal Phox and Bem1 (PB1) domain functions in the oligomerization of p62 and drives the formation of the filamentous structure, which is essential for p62 function and can enhance cargo binding efficiency (Lamark et al., 2003; Ciuffa et al., 2015). The ZZ type finger domain following the N terminal PB1 domain binds to the N-terminal arginylated protein as well as K48- and K63-linked ubiquitin chains involved in cargo recognition and interaction (Tan et al., 2014; Cha-Molstad et al., 2015; Zaffagnini et al., 2018). The LC3-interacting region (LIR, also known as AIM in yeast and plants) motif and a ubiquitin-associated (UBA) domain enables p62 to interact with both ubiquitinated cargos and LC3, which facilitates p62 to anchor the ubiquitinated cargo to autophagosome (Lamark et al., 2009).

Mammalian NBR1 is another autophagosome cargo receptor identified in mammalian cells, which shares similar domains with p62. It has been demonstrated that both p62 and NBR1 function in protein and organelle quality control (Pankiv et al., 2007; Kirkin et al., 2009), pathogen defense (Zheng et al., 2009) and cell signaling (Moscat and Diaz-Meco, 2009; Duran et al., 2011) in mammalian cells. Mammalian NBR1 has a N-terminal PB1 domain, a following ZZ domain, a LIR motif and a C-terminal UBA domain (Lamark et al., 2009). One important difference between p62 and NBR1 in mammalian cells is that p62 oligomerizes through its PB1 domain while NBR1 oligomerizes via its coil-coil domain which is absent in p62 (Lamark et al., 2009). As autophagy receptors, p62 and NBR1 have been shown to be involved in the selective autophagy of several organelles. A study in HeLa cells has shown that knockdown of either NBR1 or p62 suppresses the degradation of peroxisomes (Deosaran et al., 2013). In this study, NBR1 was shown to mediate pexophagy through binding to the ubiquitinated membrane protein on the peroxisomes surface via its JUBA domain (Deosaran et al., 2013). What’s more, p62 is suggested to cooperate with NBR1 in NBR1-mediated pexophagy via interacting with NBR1 through its PB1 domain and binding peroxisomes with its UBA domain (Deosaran et al., 2013). Besides, p62 has been shown to function in PINK1/Parkin-mediated mitophagy (Geisler et al., 2010). However, it remains to be verified whether p62 is necessary for the mitophagy, because of the conflicting results in different studies (Geisler et al., 2010; Lazarou et al., 2015).

Two plant NBR1 homologs, the Arabidopsis AtNBR1 and tobacco Joka2, have been characterized. These two proteins have been shown to share similar domain organizations and characteristics with mammalian NBR1 and p62 (Svenning et al., 2011; Zientara-Rytter et al., 2011; Zientara-Rytter and Sirko, 2014). AtNBR1 was identified as the functional hybrid of mammalian p62 and NBR1, for it shows a similar domain organization and sequence similarity with mammalian NBR1 but self-polymerizes through its PB1 domain which is more similar with p62 (Svenning et al., 2011). Biochemical data has shown it can bind ATG8 homologs with its LIR motif and interact with ubiquitin via its C-terminal UBA domains (Svenning et al., 2011). This enables it to function as an autophagy cargo receptor mediating the degradation of ubiquitinated cargos. Apart from being the autophagy substrate and the cargo receptor (Svenning et al., 2011; Huang et al., 2019), the function of plant NBR1 homologs in protein quality control and pathogen defense has also been revealed. AtNBR1 may function in the selective autophagy of protein aggregates because of the defect in vacuolar delivery of aggregation-prone GFP-FL2ΔSP observed in *nbr1* mutant (Jung et al., 2019). Studies have also shown that plant NBR1 homologs are involved in plant immunity against viruses and pathogens (Hafren and Hofius, 2017; Hafren et al., 2017; Dagdas et al., 2018). However, compared to studies on NBR1 and p62 in mammalian cells, functions of plant NBR1 are still largely unknown. For example, it is still unclear whether AtNBR1 functions in the plant abiotic stress response, as different results were obtained in different studies (Zhou et al., 2013; Rodriguez et al., 2014). Also, in contrast to the well-studied functions of mammalian NBR1 and p62 in pexophagy and mitophagy, the roles of plant NBR1 homologs in organellophagy are largely unknown. More interestingly, several recent studies have suggested that AtNBR1 is not necessary for pexophagy because the peroxisome abundance as indicated by peroxisomal marker GFP-PTS1/CFP-SKL was not affected in *nbr1* mutants vs. the WT (Jung et al., 2019; Young et al., 2019). All these data indicate the existence of different mechanisms of organellophagy in plants compared to mammals.

## Identification of Other Receptors for Selective Autophagy in Plants

To identify selective autophagy receptors responsible for the degradation of cargos, first the question whether the content change of this specific cargo was affected by autophagy should be answered. This criterion was applied in identification of the receptor which mediated the degradation of the 26S proteasome (Marshall et al., 2015). 26S proteasome, a 2.5-MDa, self-compartmentalized complex, is composed of the core protease (CP) with four stacked heptameric rings and the 19S regulatory part (RP) with two sub-complexes, base and lid. The elevated levels of 26S proteasome subunits was first noticed under normal conditions in autophagy mutants with no change of the corresponding subunit transcripts. To exclude the involvement of unassembled polypeptides, the 26S proteasome assembly analysis was conducted by fractionation and the result showed that the 26S proteasome assembled normally in autophagy mutants. In order to make sure that the subunits are representative of assembled proteasomes, the proteasome inhibitor epoxomicin with a fluorescent signal was used to track active proteasomes and the signals showed a similar pattern with these single subunits, confirming that the whole proteasomes are cleared via an autophagic pathway (Marshall et al., 2015). Similarly, in research into autophagy-dependent degradation of plastids, the first noticed phenotype was also the increased accumulation of ATI-PS bodies in cotyledon cells under carbon starvation (Michaeli et al., 2014). Another example is the abundant accumulation of the regulator BES1 in the brassinosteroid pathway in autophagy mutants which have been proved to be degraded in an autophagic manner (Nolan et al., 2017). In addition, excess starch was observed in ATG6-silenced plants under dark conditions suggesting the autophagic degradation of leaf starch (Wang et al., 2013).

Secondly, whether the cargo (e.g., 26S proteasome, ATI-PS bodies, BES1 or SSGL) is degraded by ATG8-mediated autophagy should be tested by analyzing the localization of fluorescent labeled cargo and ATG8 or measuring the cargo accumulation in autophagy mutants’ vacuoles under stress conditions. Thus, a co-localization of CP or RP subunit and autophagy marker ATG8 was observed after nitrogen starvation and concanamycin A treatment by confocal fluorescence microscopy. This was further verified by the non-accumulation of these subunits in the vacuoles of autophagy mutants *atg7* or *atg10* (Marshall et al., 2015). In other research, the transportation of ATI1-PS bodies and SSGL to vacuoles was also affected in autophagic gene-silenced plants under carbon starvation or prolonged darkness (Wang et al., 2013; Michaeli et al., 2014).

Thirdly, to assess degradation of a specific cargo more quantitatively, the autophagic flux can be measured by calculating the ratio of free GFP to the GFP-tagged parent (GFP cleavage assay) after treatment with stress inducers in autophagy mutants and the corresponding wild type. In the degradation of 26S proteasome, while exposed to nitrogen starvation, a steady decline of most 26S proteasome subunits was observed in the wild type as proteasomes were cleared (Marshall et al., 2015).

Fourthly, as autophagy receptors may bind both their targets and ATG8, an interaction between the candidate receptors and ATG8 can be analyzed by Yeast-2-Hybrid (Y2H), BiFC, Co-IP, pull-down and so on. By using Y2H and BiFC, the interaction of ATG8-ATI1 and ATG8-RPN10 was identified in the degradation of plastids and 26S proteasome, respectively (Michaeli et al., 2014; Marshall et al., 2015). The tripartite interaction of the receptor, targets, and ATG8 can be confirmed by *in vitro*-pull down assay using purified recombinant proteins or by a Calnexin-ATG8 (CNX-ATG8) recruitment assay. In the CNX-ATG8 recruitment assay, the ER-localized CNX-ATG8 will recruit receptor and its cargo to the ER via ATG8-receptor-cargo interaction. In this assay, the receptor functions as a bridge to connect the cargo with ATG8, in which three different fluorescent proteins (e.g., mCherry, YFP, and CFP) can be used to tag the receptor, cargo, and ATG8, respectively, for visualization. To be an interacting part of ATG8, the candidate receptor must have an ATG8 interacting motif (AIM). A truncated form of the receptor can be used to identify the interacting domain of receptor with AIM or targets. In the degradation of 26S proteasome, various truncations of RPN10 was used to identify its domain UIM2 which interacted with ATG8 (Marshall et al., 2015).

Lastly, to visualize the degradation of a specific cargo, cells of mutants related to autophagy and receptors expressing a specific cargo marker can be used to image the vacuolar delivery of the cargo under concanamycin A treatment or stress conditions (Michaeli et al., 2014; Marshall et al., 2015). In the mutant cells, the cargo cannot be delivered to the vacuole, and the colocalization ratio of autophagosome and cargo may decrease compared with the wild type (Michaeli et al., 2014; Marshall et al., 2015). In addition, protoplasts from the receptor mutants or RNAi plants can also be used to detect the recruitment of the specific cargo to the autophagosome. For example, BES1 failed to target to the autophagic pathway in protoplasts from DSK RNAi plants (Nolan et al., 2017).

## Challenge and Future Perspectives

Autophagy research has developed extensively in yeast and mammalian cells over the last decades, and studies on interaction and communication between autophagosomes and other organelle has proceeded at unprecedented details. However, in plants, research has been complicated largely because plants have a much higher diversity of gene families and function. It will be a challenge to figure out how things are going on in differentiated cells since most plant cells have a large central vacuole which occupies more than 90% of the cells volume. From another perspective, the most attractive part of a plant autophagy study is the unique features of the cellular system such as the vacuole and chloroplast. The energy balance in the plant is linked to autophagy. Hormone-related autophagy regulation is also a gold mine for both fundamental research and industrial applications, since hormones are pivotal to the regulation of plant development, stress resistance and immune response. In autophagosome related membrane contact studies so far, the functional tethering molecules are still missing, and it is still not clear whether mature autophagosome would form MCSs with other organelles especially the ER. New techniques have become available in plant cell biology research in recent years, such as high-resolution imaging and electron tomography, enabling the observation of potential autophagic membrane contacts.

In selective autophagy research, it is still a tough mission to identify receptors, and to figure out how receptors recognize and interact with their cargos. For example, extensive studies have reported the details of the recognition of ubiquitinated cargo by mammalian p62, however corresponding attempts in plants have not been so successful. Progress is limited by our current experimental means. Thus, overexpression of proteins is likely to cause aggregation, which is liable to affect the receptor-cargo recognition. Furthermore, how to mimic the specific/natural conditions for such studies remains laborious.

To conclude, although there is still a long way to go, plant autophagy studies are still fascinating and applicable as it may target most organelles for degradation. Undoubtedly, with continual effort and the application of new techniques and methods, we will have more clear understanding about plant autophagy interaction network in the forthcoming future.

## Author Contributions

LJ designed the concept and the organization of the manuscript. All authors wrote and edited the manuscript.

## Conflict of Interest

The authors declare that the research was conducted in the absence of any commercial or financial relationships that could be construed as a potential conflict of interest.
